# Genome sequence of a mumps virus isolated from the cerebrospinal fluid of a Vietnamese child with meningitis

**DOI:** 10.1128/mra.00375-26

**Published:** 2026-05-18

**Authors:** Xuan Duong Tran, Thi Loi Dao, Minh Mahn To, Bernard La Scola, Philippe Colson, Philippe Gautret

**Affiliations:** 1 Thai Binh University of Medicine and Pharmacy 71382, Thai Binh, Vietnam; 2 IHU Méditerranée Infection 290815, Marseille, France; 3 Aix Marseille Univ, AP-HM, SSA, RITMES, Marseille, France; 4 Aix Marseille Univ, IRD, AP-HM, MEPHI, Marseille, France; Katholieke Universiteit Leuven, Leuven, Belgium

**Keywords:** mumps, meningitis, Vietnam, vaccine strain

## Abstract

We report here the genome of a mumps virus isolate obtained by next-generation sequencing from Vero E6 cell culture supernatant of cerebrospinal fluid from a 7-year-old boy who presented with meningitis in Vietnam. It belongs to genotype N and is most closely related to mumps virus strains L-Zagreb and L3.

## ANNOUNCEMENT

Mumps is a highly contagious disease usually characterized by parotitis, with fever and other non-specific symptoms. Meningitis occurs in <10% of cases (1). The mumps virus (MuV) was discovered in 1934 and recently renamed *Orthorubulavirus parotitis* within the family *Paramyxoviridae*, subfamily *Rubulavirinae* (2). Its genome is a linear, non-segmented negative-sense, single-stranded RNA approximately 15 kilobases long. It encodes two surface glycoproteins, a fusion protein, a hemagglutinin-neuraminidase (HN), four core proteins, a nucleoprotein, a virion/phosphoprotein, a matrix protein, a large protein, and a putative membrane-associated small hydrophobic (SH) protein (3, 4). Twelve genotypes (A to N, excluding E and M) were defined based on HN- and SH-encoding genes (4), and 1,344 (near) full-length genomes (size ≥80% of complete genomes) were available in GenBank (5) as of 19 February 2025, obtained from patients sampled between 1970 and 2025, in all but five cases before 2020, from 19 countries, mostly the United States (*n* = 762) and Canada (353).

We isolated a MuV strain from the cerebrospinal fluid (CSF) of a 7-year-old male patient hospitalized in Thai Binh, Vietnam, in September 2020, with fever, bitemporal headache, fatigue, and vomiting, but no parotitis. The patient was not vaccinated against mumps. The CSF was cloudy and showed an increased white blood cell count (2,560 cells/mm³; 97% lymphocytes) and hyperproteinorachia (0.85 g/L). MuV was isolated by inoculating 500 μL of this CSF, passed through a 0.22-μm pore-sized centrifugal filter (Merck-Millipore, Darmstadt, Germany) onto Vero E6 cells (ATCC CRL-1586) (Minimum Essential Medium culture medium, 4% fetal calf serum, 1% glutamine) incubated at 37°C. A cytopathic effect was detected after 3 days.

DNA/RNA was extracted from 200 µL of culture supernatant with the MagMAX Viral/Pathogen Nucleic Acid Isolation Kit on a KingFisher Flex System (Thermo Fisher Scientific, Waltham, MA, USA). The next-generation sequencing (NGS) of the nucleic acid extract was then performed, after reverse transcription/cDNA synthesis, using Illumina technology with the Nextera XT DNA Kit and 2 × 250 bp reads on a MiSeq instrument (Illumina, San Diego, CA, USA), following manufacturer’s instructions. NGS reads (*n* = 548,510) were processed using CLC Genomics Workbench (https://digitalinsights.qiagen.com/products-overview/discovery-insights-portfolio/analysis-and-visualization/qiagen-clc-genomics-workbench/) with default parameters and mapped to MuV reference genome GenBank Accession no. OK440541.1 with criteria of >90% coverage and >80% similarity.

**Fig 1:**
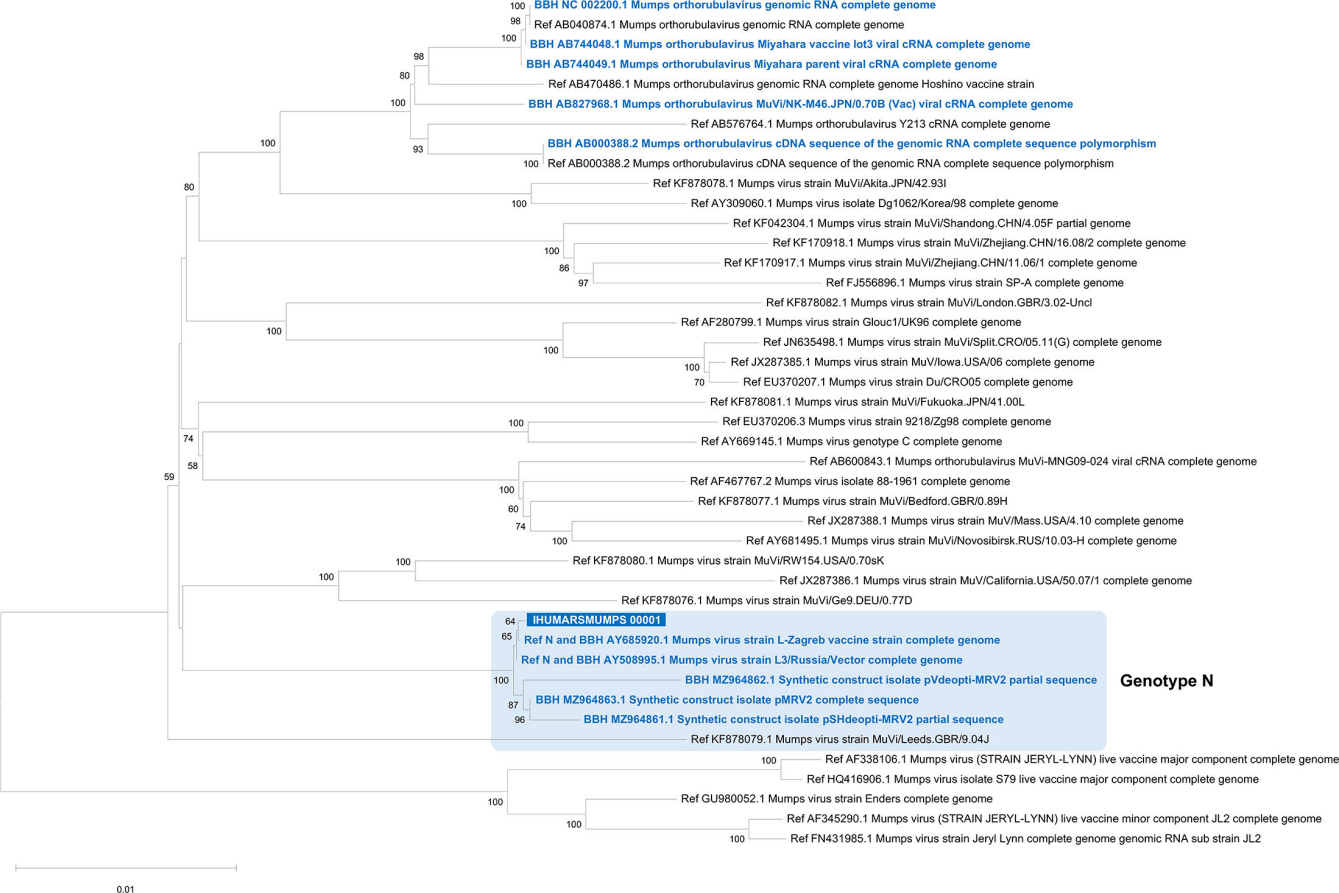
Phylogeny reconstruction based on mumps virus genomes recovered in the present study or available from GenBank. A total of 45 nucleotide sequences were analyzed, including the complete genome obtained here (indicated by a blue background and a white bold font), its 10 best BLAST hits from GenBank (indicated by a blue bold font), and reference genomes for genotypes from (4). The pairwise deletion option was applied to all ambiguous positions for each sequence pair, resulting in a final data set comprising 15,224 positions. Sequence alignment had been performed using the MAFFT v.7 web application (https://mafft.cbrc.jp/alignment/server/index.html) with automatic parameter optimization and multi-threading to improve speed and accuracy. Phylogeny reconstruction was performed using the MEGAv12 software. The evolutionary history was inferred using the neighbor-joining method, and evolutionary distances were computed using the Kimura 2-parameter method and are in the units of the number of base substitutions per site. The percentage of replicate trees in which the associated taxa clustered together in the bootstrap test (1,000 replicates) are shown next to the branches. The tree is drawn to scale, with branch lengths in the same units as those of the evolutionary distances used to infer the phylogenetic tree. The pairwise deletion option was applied to all ambiguous positions for each sequence pair, resulting in a final data set comprising 15,224 positions.

A MuV genome, 15,224 nucleotides long, was obtained. The GC content was 42.64%. Coverage and NGS depth relative to genome OK440541.1 (2017) were 98.9% (due to sequencing defects at the 5′ and 3′ ends) and 3,197, respectively. The genome was classified by Nextclade (https://clades.nextstrain.org/) and phylogeny (using MEGAv12 (https://www.megasoftware.net/) (Fig. 1) as genotype N. It exhibited 99.96% similarity to its best BLAST (https://blast.ncbi.nlm.nih.gov/Blast.cgi) hits, the genotype N genomes AY685920.1 and AY508995.1, corresponding to MuV strain L-Zagreb (vaccine strain) and L3/Russia/Vector, respectively. These findings suggest that the patient could have been infected with a vaccine strain through horizontal transmission following exposure to a vaccinated individual, as previously reported in Belarus and Croatia with the L-Zagreb mumps vaccine strain (6). Indeed, in 2020, an Indian vaccine, prepared from the L-Zagreb strain, was used in Vietnam.

Prior findings warrant intensified retrospective and prospective MuV genome sequencing to obtain a more detailed picture of the epidemiology, diversity, and evolution of this virus at the global scale.

## Data Availability

The genome sequence described here was deposited in the GenBank sequence database (https://www.ncbi.nlm.nih.gov/genbank/) under accession no. PZ133609. Next-generation sequencing raw data has been submitted to the National Center for Biotechnology Information Sequence Read Archive (https://www.ncbi.nlm.nih.gov/sra): Project accession no. PRJEB111017, experiment no. ERX16329070, run no. 16945397.
